# Prevalence of and risk factors for MRSA colonization in HIV-positive outpatients in Singapore

**DOI:** 10.1186/1742-6405-9-33

**Published:** 2012-11-06

**Authors:** Win Mar Kyaw, Linda Kay Lee, Wong Chia Siong, Angela Chow Li Ping, Brenda Ang, Yee Sin Leo

**Affiliations:** 1 Department of Clinical Epidemiology, Communicable Disease Centre, Tan Tock Seng Hospital, 11 Jalan Tan Tock Seng, Singapore, 308433, Singapore; 2 Saw Swee Hock School of Public Health, National University of Singapore, Singapore, Singapore; 3 Department of Epidemiology, School of Public Health, University of California Los Angeles, Los Angeles, USA; 4 Department of Infectious Diseases, Communicable Disease Centre, Tan Tock Seng Hospital, 11 Jalan Tan Tock Seng, Singapore; 5 Department of Medicine, Yong Loo Lin School of Medicine, National University of Singapore, Singapore, Singapore

**Keywords:** Methicillin-resistant *Staphylococcus auerus*, HIV, Risk factors, Colonization

## Abstract

**Background:**

Whilst there have been studies on the risks and outcomes of MRSA colonization and infections in HIV-positive patients, local data is limited on the risk factors for MRSA colonization among these patients. We undertook this study in a tertiary HIV care centre to document the risk factors for colonization and to determine the prevalence of MRSA colonization among HIV-positive outpatients in Singapore.

**Methods:**

This was a cross-sectional study in which factors associated with MRSA positivity among patients with HIV infection were evaluated. A set of standardized questionnaire and data collection forms were available to interview all recruited patients. Following the interview, trained nurses collected swabs from the anterior nares/axilla/groin (NAG), throat and peri-anal regions. Information on demographics, clinical history, laboratory results and hospitalization history were retrieved from medical records.

**Results:**

MRSA was detected in swab cultures from at least 1 site in 15 patients (5.1%). Inclusion of throat and/or peri-anal swabs increased the sensitivity of NAG screening by 20%. Predictors for MRSA colonization among HIV-positive patients were age, history of pneumonia, lymphoma, presence of a percutaneous device within the past 12 months, history of household members hospitalized more than two times within the past 12 months, and a most recent CD4 count less than 200.

**Conclusions:**

This study highlights that a proportion of MRSA carriers would have been undetected without multiple-site screening cultures. This study could shed insight into identifying patients at risk of MRSA colonization upon hospital visit and this may suggest that a risk factor-based approach for MRSA surveillance focusing on high risk populations could be considered.

## Background

Methicillin-resistant *Staphylococcus auerus* (MRSA) infections present a significant burden in terms of morbidity, length of hospitalization, and rising healthcare costs [1-3]. Studies have shown that staphylococcal colonization is a predominant risk factor for subsequent infection, and nasal carriage of *Staphylococcus aureus* increases the risk of subsequent infection two- to twelve-fold, especially during hospitalization [4-7]. Risk factors associated with MRSA colonization include frequent exposure to healthcare settings, previous MRSA infections, and frequent antibiotic usage [8-13].

HIV-positive patients are at higher risk of MRSA colonization associated with frequent exposure to healthcare facilities, frequent oral antibiotic intake, low CD4 count, and other behavioral risk factors [13-15]. HIV has been recognized as an independent risk factor for colonization with MRSA [16], and *S. aureus* infections are known to be responsible for substantial morbidity and mortality in HIV-positive patients [17,18].

Whilst there have been studies on the risks and outcomes of MRSA colonization in HIV-positive patients, local data is limited on the risk factors for MRSA colonization among these patients. We undertook this study in a tertiary HIV care centre to document the risk factors for colonization and to determine the prevalence of MRSA colonization among HIV-positive outpatients.

## Methods

### Study type and study population

This was a cross-sectional study in which factors associated with MRSA positivity among patients with HIV infection were evaluated. The study was done at the Communicable Disease Centre (CDC) at Tan Tock Seng Hospital (TTSH), the national referral centre for HIV. CDC is administratively responsible to TTSH, the second largest general hospital in Singapore, with 1,200beds. All HIV-positive patients who attended the specialist outpatient clinic at CDC between 12^th^ November and 1^st^ December 2008 were asked to participate. Verbal consent was obtained from all participants.

### Data sources and data collection

A set of standardized questionnaire and data collection forms were available to interview all recruited patients. Information collected include visits to a healthcare facility in the past 12 months, contact with healthcare workers (HCW) in the past 6 months, hospitalization history of household members, presence of a percutaneous device in the past 12 months or urinary catheter in the past 6 months; surgery in the past 12 months, and use of intravenous and oral antibiotics in the past 1 month and 3 months, respectively. This information was collected through interviews by a research staff.

Following the interview, trained nurses collected swabs from the anterior nares, axilla, groin, throat, and peri-anal regions. The first three areas were swabbed using a single swab stick. Separate swab sticks were used for the throat and peri-anal region. Samples were inoculated on chromogenic agar plates (MRSA*Select*, BioRad, France) and incubated at 37°C for 18–28 hours. Growth of pink or mauve colonies was read as MRSA positive, while colorless colonies were MRSA negative.

Information on demographics, clinical history, laboratory results and hospitalization history were retrieved from medical records. These included the presence of co-morbidities, admission to intensive care unit or general hospitalization in the past 12 months, use of antiretroviral (ART) therapy during the preceding 12 months, and current usage of co-trimoxazole and beta lactam antibiotics in the past six months. The most recent CD4 count during the preceding 6 months and previous MRSA colonization history were also collected.

### Statistical analysis

For this prospective case control study, cases were defined as HIV-positive patients with laboratory-confirmed MRSA colonization, and controls as HIV-positive patients without laboratory-confirmed MRSA colonization within the study period.

Cases and controls were compared by univariate analysis to determine differences in the allocation of potential risk factors. Descriptive statistics were used to describe the distribution of the variables in the study population. A chi square test or Fisher’s exact test was used to evaluate differences in categorical variables. Crude odds ratios for continuous variables were obtained by using logistic regression. All variables with p<0.05 in univariate analysis were included in multivariate analysis to identify independent risk factors of MRSA. A stepwise logistic regression model was used to select variables for inclusion in the final model. Variables with collinearity were not simultaneously considered in the final model.

### Study approval

This study was approved by the domain specific review board (DSRB) of the National Healthcare Group, Singapore (DSRB Reference No. 2009/00545).

## Results

### Descriptive statistics and univariate analysis

A total of 296 HIV-positive patients were recruited, giving us a response rate of 93%. They were predominantly male (n=257, 86.8%), and the median age of these patients was 43 years (range, 18–73). Among the 296 patients, MRSA was detected in swab cultures from at least 1 site in 15 patients (5.1%). Out of these 15 patients, 6 had positive MRSA cultures from their nasal/axilla/groin swab only while 3 were positive in either throat or peri-anal swabs only (Table 1). The demographic characteristics of the 15 cases and 281 controls are shown in Table 2. There was a slight male predominance among the case patients; males were 2.2 times more likely than females to have MRSA.

**Table 1:** **Sites of Methicillin-resistant *Staphylococcus aureus *(MRSA) colonization**

**Site**	**MRSA Position Cases [n=15]**
NAG only	6
Throat only	2
Peri-anal only	1
NAG + Throat	2
NAG + Peri-anal	1
NAG + Throat + Peri-anal	3

NAG = Nares, axilla, groin.

**Table 2:** Risk factors for MRSA colonization: Univariate analysis

**Vañable**	MRSA Position n (%)*	MRSA Negatve n (%)*	**OR (95% CI)**	**P value**
**Demographics**				
Age(median, range), years	50 (30–67)	43 (18–73)	1.1 (1.00-1.11)	0.036
Age>=65	1 (6.7)	7 (2.5)	2.8 (0.32-24.32)	0.352
Gender, Male	14 (933)	243 (86.5)	2.2 (0.28-17.13)	0.455
AIDS-related infections				
Tuberculosis	3 (20.0)	46 (16.4)	1.3 (0.35-4.71)	0.713
Pneumoncystic jiroveci pneumonia	8 (53.3)	73 (26.0)	3.3 (1.14-9.30)	0.027
Bacterial Pneumonia	4 (26.7)	24 (8.5)	3.9 (1.15-13.17)	0.029
Cytomegalovirus infection	3 (20.0)	25 (8.9)	2.6 (0.68-9.68)	0.166
Skinlesion	6 (40.0)	70 (24.9)	2.0 (0.69-5.85)	0.200
Lymphoma	3 (20.0)	4 (1.4)	17.3 (3.48-86.15)	<0.001
Toxoplasmosis	1 (6.7)	6 (2.1)	3.3 (0.37-29.08)	0.287
**Exposure to Health Facilities**				
Prior hospitalization in the past one year	9 (60)	44 (15.7)	81 (274–23.84)	<0.001
Outpatient visit to CDC in the past one year	15 (100)	259 (92.2)	NC	0.260
Admission to Intensive care unit in the past one year	1 (6.7)	1 (0.4)	20.0 (1.19-336.64)	0.038
Polyclinic visit in the past one year* *	7 (46.7)	125 (44.5)	1.1 (0.39-3.09)	0.870
Surgery in the past one year* *	1 (6.7)	11 (3.9)	1.8 (0.21-14.55)	0.603
Presence of Percutaneous Device in the past one year* *	11 (73.3)	47 (16.7)	13.7 (4.18-44.84	<0.001
Urinary catheterization in the past six months* *	1 (6.7)	4 (1.4)	5.0 (0.52-47.22)	0.165
Household member hospitalized more than two times in the past one year * *	5 (33.3)	28 (10.0)	4.5 (1.44-14.16)	0.010
Patient contact with a health care worker in the past 6 month * *	5 (33.3)	29 (10.3)	4.3 (l.39-13.59)	0.012
Drug history				
Recent intravenous antibiotic usage	8 (72.7)	37 (78.7)	0.7 (0.16-3.23)	0.669
Oral antibiotic usage in the past three months	11 (73.3)	96 (34.2)	5.3 (1.64-17.09)	0.005
On antiretroviral therapy in the preceding one year	14 (93.3)	238 (84.7)	2.5 (0.32-17.74)	0.376
Current use of Co-trimoxazole	4 (26.7)	77 (27.4)	1.0 (0.29-3.12)	0.950
Beta lactam antibiotic usage in the past six months	5 (33.3)	10 (3.6)	13.6 (3.90-47.07)	<0.001
**Others**				
History of previous MRSA colonization	2 (13.3)	7 (2.5)	6.0 (1.14-31.89)	0.035
Most recent CD4<20O	9 (60)	71 (25.3)	4.4 (1.53-12.90)	0.006

*Unless otherwise indicated.

* * Data collected by questionnaire.

NC = not calculable.

Significant HIV-associated conditions among cases included *Pneumocystis jiroveci* pneumonia (PCP) and bacterial pneumonia. Patients with PCP were more likely to have MRSA than those without (odds ratio [OR], 3.3; 95%CI, 1.14-9.30); those with bacterial pneumonia were more likely to have MRSA as well (OR=3.9; 95%CI, 1.15-13.17). Patients with lymphoma were 17.3 times more likely than patients without lymphoma to be colonized with MRSA (95%CI, 3.48-86.15).

Among MRSA positive patients, 9 (60%) had prior hospitalization in the past year compared with 44 (15.7%) of the MRSA negative controls. HIV-positive patients who had outpatient visit(s) to CDC or a polyclinic within the past year were more likely to be colonized with MRSA compared to those who did not. However, this was not statistically significant. Additionally, those who had a percutaneous device in the past 12 months, a history of household members hospitalized more than two times in the past 12 months, or contact with a health care worker in a hospital in the past 6 months were significantly more likely to have MRSA than patients without these exposures (Table 2).

Risk factors relating to drug exposure were also evaluated in Table 2. Patients with oral antibiotic use during the last 3 months were 5.3 times more likely than patients without recent oral antibiotic usage to have MRSA (73.3% vs. 34.2%; 95%CI, 1.64-17.09). In addition, patients who used beta lactam antibiotics within the last 6 months were also more likely to be cases than patients who did not. Patients with a recent CD4 count less than 200 were 4.4 times more likely than patients with a recent CD4 greater than 200 to be cases than controls.

### Multivariate analysis

Upon multivariate analysis, predictors for MRSA colonization among HIV-positive patients were age, history of bacterial pneumonia, lymphoma, presence of a percutaneous device within the past 12 months, history of household members hospitalized more than two times within the past 12 months, and a most recent CD4 count less than 200 (Table 3). We generated a receiver operating characteristic (ROC) curve to evaluate the final logistic regression model. The area under the ROC curve (AUC) was 0.89 (95% CI, 0.83-0.97).

**Table 3:** Independent Risk Factors Associated with MRSA colonization: Multivariate analysis

**Expore**	**OR (95% CI)**	**P value**
Lymphoma	15.7 (2.0-123.10)	0.009
Most recent CD4<200	9.1 (1.187-44.53)	0.006
Hoousehold member hospitalized more than two times in the past one year	8.4 (1.80-39.18)	0.007
Presence of Percutaneous Device in the past one year	8.1 (2.07-31.81)	0.003
Pneumonia	6.5 (1.30-32.55)	0.023
Age (years)	1.1 (1.01-1.13)	0.033

## Discussion

Singapore first isolated MRSA in the early 1980s [19]. The incidence and prevalence of MRSA infections have remained steady during the last two decades in major academic medical centers, although methicillin resistance appeared in 35% of all *S. aureus* isolates in one study [20]. The incidence of HIV infection in Singapore has increased rapidly over the last decade [21].

A previous study at CDC showed that the prevalence of MRSA colonization in HIV-positive outpatients was higher than the prevalence of MRSA colonization in other populations [15]. A study by Villacian et al. using only nasal swab reported a lower point prevalence of MRSA colonization (3% vs. 5.1% in our study) and 20% colonization with methicillin-sensitive *Staphylococcus aureus* (MSSA) [15]. Most studies and screening programs evaluating the prevalence of MRSA colonization have utilized swabs from the nares, axilla and groin [22,23]. Mertz et al. have shown that the inclusion of a throat swab increased the sensitivity of detection among *S. aureus* carriers by 25.7% [22].

In this study, we showed that inclusion of throat and/or peri-anal swabs increased the sensitivity of NAG screening by 20%. The inclusion of additional swabs could be especially important for HIV positive patients, as unrecognized carriers can serve as reservoirs for transmission during frequent hospital visits. Chow et al. found that the inclusion of additional perianal and throat swabs increased MRSA detection by 12.5% in HIV positive patients [24].

Our finding that recent diagnosis of lymphoma was associated with MRSA colonization could be explained by the increased likelihood for lymphoma in advanced HIV infection when there is a greater extent of immunosuppression [25-27]. Some studies have shown an association between MRSA colonization and cutaneous T-cell lymphoma but not among HIV patients [28,29]. Similarly, we identified low CD4 count as a risk factor for MRSA colonization; this has been described in other studies among HIV-positive patients [13,30,31].

Although univariate analysis showed an association between MRSA colonization and hospitalization within the past year, this effect was negated in multivariate analysis. Recent hospitalization has been frequently described as a predictor for MRSA colonization among HIV-positive patients in the published literature [14,15]. Interestingly, in this study, we found a strong association between MRSA colonization and previous hospitalization history of household members. Studies have shown that family members of colonized patients are at risk for MRSA colonization and subsequent MRSA infection for many months [7,32,33].

The association between MRSA colonization and the presence of a percutaneous device within the past year in this study was significant. Onorato and colleagues found a seven-fold increased risk between the insertion of a central venous catheter and MRSA colonization among HIV-positive patients [14]. Both findings highlight the importance of aseptic insertion and appropriate care of such devices and may reflect that patients with indwelling devices could have more contacts with healthcare workers, making them more susceptible to MRSA.

Our observation that receipt of ARV within the past year was not associated with decreased risk of MRSA colonization is inconsistent with previous studies [31]. We found strong association between age and MRSA colonization. Studies have shown that elderly age (>65 years) is a risk factor for MRSA colonization despite patient group [24,34]. AUC value in our study showed that predictors in the final model comprising six variables can differentiate well between MRSA colonizers and non-colonizers.

Our study had some limitations. Our population was predominantly male, and the sample size was small; more independent risk factors may have been identified with a larger study population. As our data were collected at a single public hospital for only outpatients, it may not reflect all HIV-infected patients. We may have underestimated prior hospitalizations, as data collection for this study did not account for admission to other hospitals. Data collection was also limited for some risk factors previously associated with MRSA colonization in HIV infected patients such as prior incarceration and high risk sexual behaviors [35,36]. Many healthcare related factors assessed by the questionnaire in this study may be subjected to recall and reporting bias. However, this study highlights that a proportion of MRSA carriers would have been undetected without multiple-site screening cultures. HIV-positive patients are at increased risk of MRSA colonization due to immunosuppression and also their exposure to healthcare facilities.

## Conclusions

This study highlights that a proportion of MRSA carriers would have been undetected without multiple-site screening cultures. This study could shed insight into identifying patients at risk of MRSA colonization upon hospital visit. Predictors for MRSA colonization among HIV-positive patients were age, history of bacterial pneumonia, lymphoma, presence of a percutaneous device within the past 12 months, history of household members hospitalized more than two times within the past 12 months, and a most recent CD4 count less than 200. This may suggest that a risk-factor based approach for MRSA surveillance focusing on high risk populations could be considered.

### Study approval

This study was approved by the domain specific review board (DSRB) of the National Healthcare Group, Singapore (DSRB Reference No. 2009/00545).

## Abbreviations

ARV: Antiretroviral; AUC: Area under the ROC curve; CDC: Communicable Disease Centre; HCW: Healthcare workers; MRSA: Methicillin-resistant *Staphylococcus aureus*; MSSA: Methicillin-sensitive *Staphylococcus aureus*; PCP: *Pneumocystis jiroveci* pneumonia; TTSH: Tan Tock Seng Hospital.

## Competing interests

The authors declare that they have no competing interests.

## Authors’ contributions

ACLP participated in study design and data acquisition. LKL, WCS, YSL and BA participated in manuscript writing. WMK performed data acquisition, data analysis and manuscript writing. All authors read and approved the final manuscript.
